# Purely Translational Realignment in Grid Cell Firing Patterns Following Nonmetric Context Change

**DOI:** 10.1093/cercor/bhv120

**Published:** 2015-06-05

**Authors:** Elizabeth Marozzi, Lin Lin Ginzberg, Andrea Alenda, Kate J. Jeffery

**Affiliations:** 1 Department of Experimental Psychology, University College London, London WC1H 0AP, UK; 2 Department of Cell and Developmental Biology, University College London, London WC1E 6BT, UK; 3 Department of Electrical and Electronic Engineering, Imperial College London, London SW7 2AZ, UK

**Keywords:** context, grid cells, place cells, sensory integration, spatial learning

## Abstract

Grid cells in entorhinal and parahippocampal cortices contribute to a network, centered on the hippocampal place cell system, that constructs a representation of spatial context for use in navigation and memory. In doing so, they use metric cues such as the distance and direction of nearby boundaries to position and orient their firing field arrays (grids). The present study investigated whether they also use purely nonmetric “context” information such as color and odor of the environment. We found that, indeed, purely nonmetric cues—sufficiently salient to cause changes in place cell firing patterns—can regulate grid positioning; they do so independently of orientation, and thus interact with linear but not directional spatial inputs. Grid cells responded homogeneously to context changes. We suggest that the grid and place cell networks receive context information directly and also from each other; the information is used by place cells to compute the final decision of the spatial system about which context the animal is in, and by grid cells to help inform the system about where the animal is within it.

## Introduction

The entorhinal–hippocampal network forms the core of a spatial memory system that supports cognitive processes such as navigation and episodic memory. While hippocampal place cells exhibit focal, sparse, and irregular activity patches (firing fields) on an open-field arena, entorhinal grid cells show spatially regular firing field arrays (Hafting et al. 2005). Both place and grid cells position their firing fields with the aid of spatial cues such as local environmental boundaries. However, place cells also respond to nonmetric information (Wood et al. 2000; Anderson and Jeffery 2003; Leutgeb et al. 2005), and responses to metric and nonmetric inputs can be manipulated independently (Jeffery and Anderson 2003); these so-called contextual inputs are thus segregated until quite late in the processing pathway. The aim of the present experiment was to determine whether grid cells also respond to nonmetric contextual cues, and thereby gain insights into where and how the convergence of metric and nonmetric signals occurs.

A previous study of grid cells found that, while metric environmental change induced translation and sometimes rotation of grids, nonmetric change (to box color) caused no shift of grids (Fyhn et al. 2007), suggesting insensitivity to nonmetric inputs. However, those contextual changes did not induce “global remapping” (Leutgeb and Leutgeb 2007) of hippocampal place cells, and so may not have been salient enough to trigger responding by the grid cell system. In cases where grids changed their relationships to the local boundaries, spatial changes to the environment had also occurred (e.g., shift of the apparatus to a new room, or induction of darkness). It thus remains possible that grid cells would be entirely insensitive to purely nonmetric changes to the environment. To investigate this issue, we employed a nonmetric context-change paradigm known to induce place cell remapping (Anderson and Jeffery 2003), and measured changes in grid cell firing. We wanted to know (a) whether grid cells would “remap” (realign) to nonmetric change, and (b) if so, whether they would do so coherently (all in the same way) or individually, and (c) whether they could respond to unique configurations of color and odor, in the way that place cells do.

**Figure 1.**
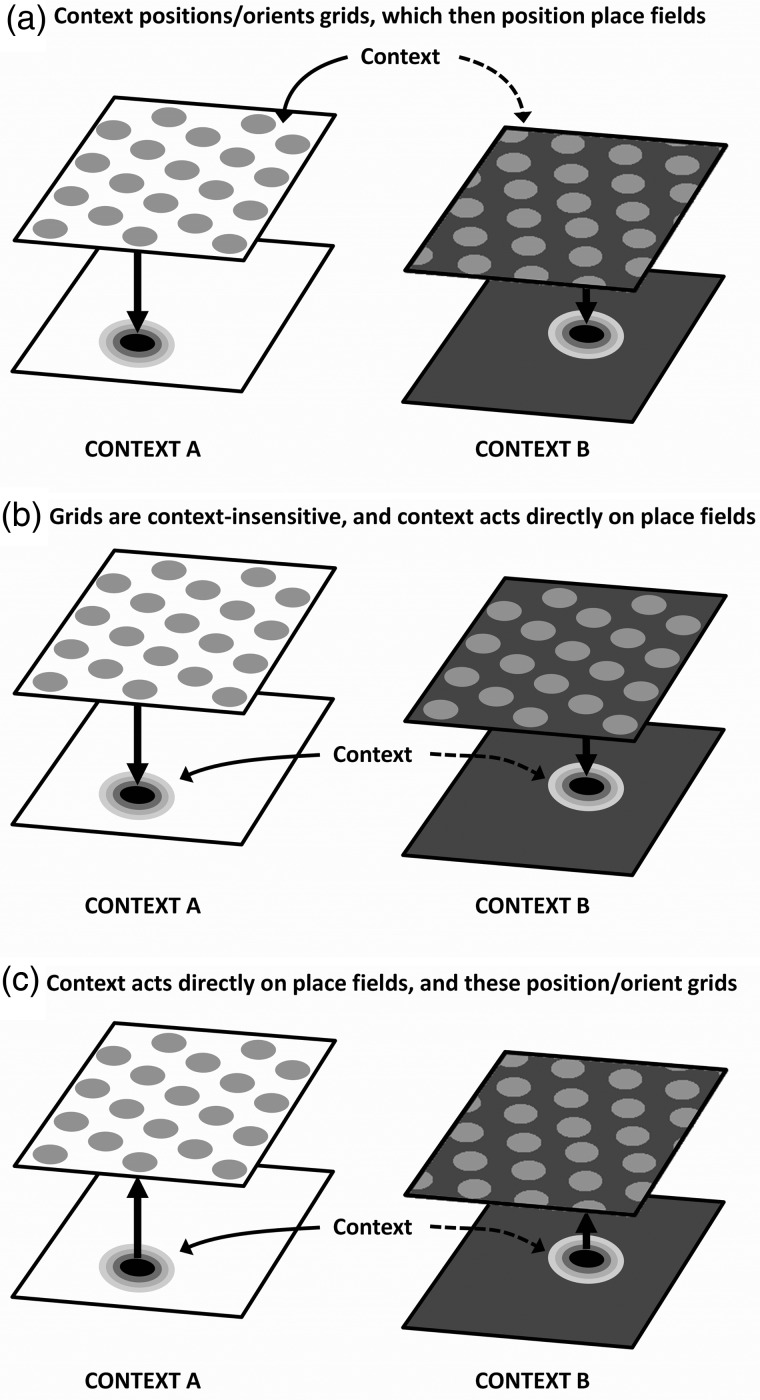
Hypotheses concerning relationships between context inputs, grid cells, and place cells. (*a*) The context signal is routed through the entorhinal grid cells and acts by regulating the position/orientation of the grids, which in turn regulate where place fields are positioned via a feedforward mechanism. (*b*) Grid cells are insensitive to context and the context signal acts directly on place cells (perhaps by gating the entorhinal input). (*c*) The context signal acts directly on place cells which feed the computed output back to the grid cell network, thus positioning the grids; grid cells are thus context sensitive, but are the consequence rather than cause of place cell context sensitivity.

At the outset, we had 3 hypotheses about the possible relationship between grid cells, place cells, and context (Fig. 1). The first is that grid cells may be fully responsive to context changes even in the case where no metric changes were made to the environment, and these responses may drive place cell remapping (supported by Fyhn et al. (2007)). Since place cell remapping in these conditions is “partial” (not all cells respond to every change; Anderson and Jeffery 2003), it is of interest to observe whether grid cell firing might also be partial, although this seems *a priori* unlikely given the documented attractor dynamics of the system (Yoon et al. 2013). The second hypothesis is that grid cells may be entirely insensitive to context, with place cells receiving context information directly from some other source, which could act by gating the entorhinal feedforward projections (Hayman and Jeffery 2008). And finally, it may be that place cells receive context information directly, and feed this back to the grid cells; a hypothesis supported by developmental work (Langston et al. 2010; Wills et al. 2010) and inactivation studies (Brandon et al. 2011, 2014; Bonnevie et al. 2013).

## Materials and Methods

### Subjects

Eighteen male Lister Hooded rats (Charles River, UK; 300–350 g) were implanted with tetrodes aimed at the medial entorhinal cortex alone (MEC; *n* = 13) or both MEC and hippocampal CA1 (*n* = 5, the hippocampal data are not reported here). Fourteen rats were recorded in small context boxes (see below) and 7 in large, with 3 animals recorded in both. After implantation, animals were housed singly and their food restricted to 90% of their free-feeding weight. Experiments were conducted according to the UK Animals (Scientific Procedures) Act 1986.

### Apparatus

**Figure 2.**
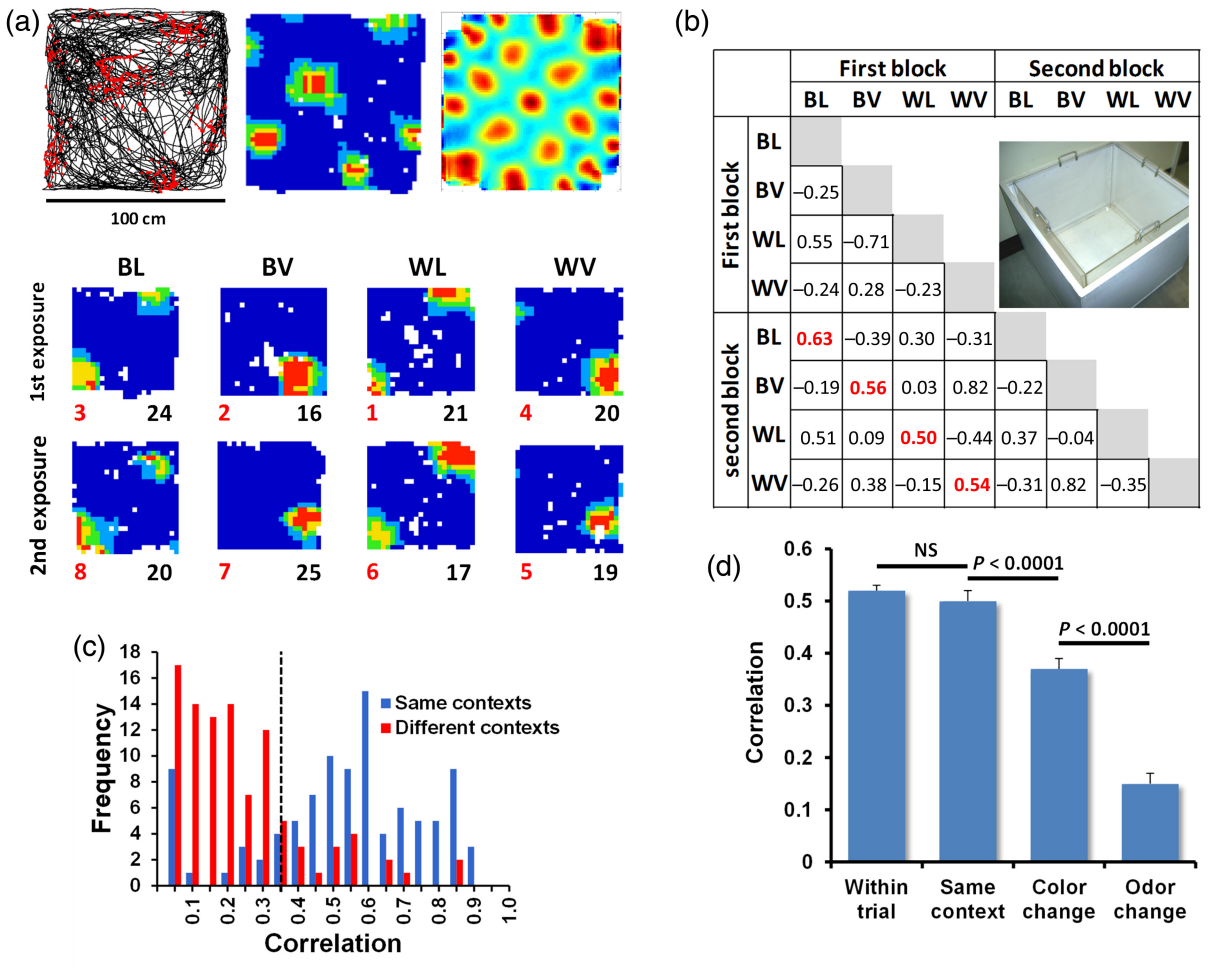
Grid cell realignment following nonmetric context change. (*a*) Top: L-R, a spike plot showing the rat's path in black and the cell's action potentials as red dots; same data plotted as a ratemap; autocorrelogram. Bottom: firing pattern of the same cell in the 4 small contexts (each run twice). BL = black-lemon, BV = black-vanilla, WL = white-lemon, WV = white-vanilla. Red numbers to show trial order, black numbers show firing rate in Hz. (*b*) Pairwise correlations of the trials in (*a*) showing how the typical remapping patterns seen in the ratemaps corresponded to a numerical correlation value. Inset shows the small white context box. The correlation matrix shows the ratemap correlation values collected for 2 blocks of 4 trials. Each trial type has been compared with each of the 2 trials of the other types, as well as with its counterpart in the other block (shown in red). Cases where clear remapping was evident visually (e.g., black-vanilla and white-lemon) generated low correlation values. (*c*) Grid ratemap correlations for same-context and different-context comparisons, showing preponderance of low correlations (realignment) in the latter. Dotted line shows the categorical realignment threshold. (*d*) Correlations for the different ratemap comparisons, including the within-trial comparisons between first and second trial-halves. Sensitivity (decreased correlation) was higher to odor change than color change.

For the small-box trials, the apparatus used was the same as that used previously in Anderson and Jeffery (2003). This comprised 2 transparent acrylic boxes 60 × 60 cm square with walls 50 cm high (Fig. 2), each wiped repeatedly throughout the experiment with either lemon or vanilla food flavoring. These inserts could then be placed into one of two slightly larger wooden boxes, one painted black and the other white. This allowed the apparent color of the boxes to change, creating 4 compound contexts: black-lemon, black-vanilla, white-lemon, and white-vanilla. For the large-box trials, the boxes (also acrylic) were 120 × 120 cm square with walls 50 cm high. Because these enclosures were too large to allow wooden casings to be manipulated, color changes were induced using 4 wooden panels painted white or black which were placed against each wall of the box, with a black or white sheet used to make the color of the floor.

Before each trial, the inner surface of the floor of the box was cleaned with tissues dampened with a small amount of ethanol and scented with lemon or vanilla food flavoring (Supercook, UK) by placing the flavoring (0.5 mL for the small boxes, 2.0 mL for the large) onto a clean paper towel and wiping the floor of the box as uniformly as possible. Context boxes were always placed in exactly the same position within the laboratory room, with all distal cues in the testing room (such as recording equipment and shelves) remaining in the same positions and available for the rat to see at all times. The box not in use during a trial was placed against the wall of the experimental room out of view of the rat.

### Electrode Implantation

Unit recordings were made using bundles of 4 tetrodes loaded into an Axona microdrive (Axona Ltd, St Albans, UK), using methods described previously (Barry et al. 2007). Recordings were made from unilateral posterior cortical sites (coordinates from lambda: 1.2 mm AP, 4–4.5 mm medio-lateral; angled 0–8° from vertical; *n* = 14).

### Recording Procedure

Animals were brought into the recording room individually in a covered carrying box, and were then removed, connected to the recording equipment (DacqUSB, Axona Ltd, St Albans, UK) via a headstage and 3-m fine cable, and placed on a holding platform. Extracellular potentials were recorded from each of the electrodes and the signal was amplified (8000–38 000 times) and bandpass filtered (500 Hz to 7 kHz). Each channel was sampled at 50 kHz and action potentials were stored at 50 points per channel whenever the signal exceeded a user-defined threshold (0.2 ms prethreshold and 0.8 ms post-threshold, total 1 ms). Each of the four wires of one tetrode was referenced to the signal from a wire on another tetrode of the same microdrive. The headstage carried 1 or 2 different-sized light-emitting diodes, the positions of which were recorded via an overhead camera to monitor position and head direction. Spike events, local field potentials, and positional information were recorded and stored for offline analysis.

If a putative grid cell was found during a screening session (usually conducted on a larger arena in a different room), then the animal was moved into the experimental room, connected to recording equipment, and placed on a holding platform, where it rested in between recording trials. It was then subjected to the experimental protocol, comprising a sequence of foraging trials in different configurations of the contexts. During each trial, rice was scattered randomly into the environment to ensure even spatial sampling, while spike and position data were collected. Each recording trial lasted 10 min (for the small-box trials) or 15 min (for the large-box trials). Between trials, animals were returned to the holding platform for a few minutes while the apparatus was reconfigured. The order of the trials was varied throughout the experiment, such that every context was experienced at least once, and same-context trials were never consecutive. For 13 rats, the series of 4 contexts was followed by a single repeated trial of one of the conditions randomly selected; for the other 5 rats, the entire sequence of 4 conditions was repeated, in a different order. Animals that were run in both the small and large context box experiments were run in each experiment in a pseudorandom fashion with at least 4 h between the recordings.

### Data Analysis

Spike data were analyzed offline using a cluster-cutting program (Tint; Axona Ltd, St Albans, UK) followed by analysis in Matlab (The MathWorks, Natick, MA, USA). Units were isolated manually or with the help of an automated clustering algorithm (KlustaKwik; Kadir et al. 2014). Spatial firing was assessed by plotting the spikes on the path of the rat; cells that appeared to show spatial firing visually were then subjected to a series of analyses to determine whether they would be included in the final dataset. Cells were accepted into further analysis if at least one of their between-trial ratemap correlations (see below) was >0.49. Cells were considered grid cells if they were 1) recorded on MEC-targeted electrodes, and 2) if at least one of their grid scores, in screening or context trials, was >0. We treated the data from the small- and large-box trials separately; within these datasets, cells were considered to be repeat instances, and hence discarded, if they 1) occurred on the same tetrode, 2) had similar waveforms (i.e., their maxima occurred on the same electrode), and 3) were recorded within 10 days of the previous recording in the same box type. Where more than one grid cell cluster occurred on the same tetrode, we checked that these were unlikely to be from the same cell by first correlating their 2 ratemaps on a black-vanilla trial, and then, if the correlation exceeded 0.3 (which was the case for 6 cell pairs), checking that the clusters were nevertheless well separated in the cluster plot.

### Statistical Analysis

Firing characteristics were analyzed as described below. Parametric comparisons of these between conditions used *t*-tests, analysis of variance (ANOVA) (with post hoc tests Bonferroni corrected) and *χ*^2^ analyses.

### Ratemap Generation

Locational firing rate maps were made by binning data in 2 × 2 cm bins, dividing by dwell time, and smoothing with a 5-bin boxcar filter.

### Firing Rate Analysis

The peak firing rate for a trial (in Hz) was the firing rate in the highest bin. To assess changes in firing rate, a rate difference score between 2 recording trials was calculated (Leutgeb et al. 2005), being the absolute difference between the rates divided by their sum.

### Spatial Correlations

Spatial correlations between pairs of ratemaps were computed by calculating a Pearson's *r* correlation between firing rates of homologous pixels in the 2 smoothed maps. Unvisited bins as well as bins having zero firing in both maps were excluded from this calculation in order to prevent artificially high correlations. Grid cells were only entered into the final analysis if at least one of their trial-pair correlations in the trial series was >0.49, this value being the 95th percentile of a distribution derived from correlating ratemaps chosen randomly from pairs of co-recorded cells. A frequency histogram comparing the distributions of same-trial versus different-trial correlations revealed a maximal separation point at 0.35 (Fig. 2*b*) and so this value was used as the threshold for categorical comparison of realigning versus nonrealigning cell numbers.

### Extracting Grid Metrics

Spatial autocorrelograms of the smoothed ratemaps were generated and used for assessment of the scale and orientation of grids (Hafting et al. 2005; Sargolini et al. 2006; Barry et al. 2007). The cross-correlogram was generated in the same way as the autocorrelograms, except that a given map was correlated against another instead of itself.

### Grid Score

Periodic grid firing fields (Sargolini et al. 2006) were calculated as follows: a circular region was defined, the radius of which was the furthest distance from the center of the spatial autocorrelogram to the center of one of the six peaks. This circular region was then extracted and a copy rotated in 30° increments up to 180° (5 rotations) and re-correlated each time with the original. Due to the hexagonal periodic firing of grid cells, the correlations between the original and rotated copy vary sinusoidally with highest correlations occurring at rotations of 60°, 120°, or 180° and lowest at rotations of 30°, 90°, or 150°. The difference between the highest and lowest correlation peak comprised the grid score. The grid score was calculated from the large-arena screening trials where these were available (*n* = 79 cells) or from the recording trials if screening trials were unavailable (*n* = 21), and cells were rejected (*n* = 3) if none of their scores exceeded 0.

### Grid Scale

Grid scale was obtained from the autocorrelograms generated from the ratemaps in the large context boxes, by taking, for each trial, the mean of the distance (in bins) of the 6 closest peaks to the central peak. The difference in grid scale between 2 trials was expressed as a difference score, calculated as the difference between the scales divided by their sum. Scores were computed for trial pairs of the same condition, trial pairs of different conditions for the same cell, and trial pairs between randomly selected cells obtained when the entire dataset was shuffled; these were compared using one-way ANOVA.

### Grid Orientation

Grid orientation was calculated from the autocorrelograms. Orientation was defined as the angle between the *x*-axis and a line connecting the center of the autocorrelogram to the center of the nearest peak encountered anticlockwise from the positive *x*-axis. Average change in orientation for a given set of 5 trials was derived by calculating the absolute (unsigned) change in orientation between each trial pair and averaging. These values were compared against trial-pair differences for the repeated trials (in which orientation differences should be small) and for the same grid set shuffled (in which differences should be larger, although not as large as those chosen from a random distribution due to additional environmental influences on grid orientation (Stensola et al. 2012)).

### Phase Shift

The amount the fields of a grid were shifted relative to the box walls following a context change was calculated from the cross-correlogram (Hafting et al. 2005) of the ratemaps of the 2 trials to be compared. The phase shift (i.e., the distance translocated by grid fields) was then calculated as the distance (in bins) from the center of the peak closest to the center of the cross-correlogram.

### Ensemble Analyses

To derive an estimate of whether cells reacted together, or as individuals, to a given change, we performed ensemble analyses on sets of simultaneously recorded cells. A given value, being either ratemap correlation or phase shift, was generated for each cell for a given trial pair, and the resulting values then correlated between that cell and every other cell in the ensemble. The resulting set of numbers was then subjected to a Pearson's *r* analysis.

### Histology

At the end of the experiment, rats were deeply anesthetized and perfused with saline followed by a 4% paraformaldehyde solution (PFA), and the brains removed and stored in a solution of 4% PFA and 20% sucrose prior to sectioning. Hemispheres that had received the entorhinal implant were cut sagittally at 40 μm intervals. Slices were mounted on gelatin-coated slides and stained with cresyl violet. The depth and cell layer were ascertained by pairing visual information from the microscope with estimates of the distance that the electrodes were moved during recording.

## Results

In total, 124 spatially firing neurons were recorded; the selection criteria (see Materials and Methods) collectively resulted in exclusion of 26 neurons, leaving 98, and produced a dataset that looked visually to include grid cells and not to include nongrid cells. Histology showed that the grid cells were located in parasubiculum and MEC (see Supplementary Figs 1–4); an example of a screening trial from each of the rats is shown in Supplementary Figure 5, confirming that the cells from these regions were indeed grid cells. Of the 98 cells in the final dataset, 71 were from superficial layers, 26 were from deeper layers, and 2 could not be classified. There were no apparent differences between the responses of grid cells from the different layers and so all cells were analyzed together.

### Response of Grid Cells to Context Change

We first looked to see whether grid cells changed their firing rates in response to context change, by comparing same-context and different-context trial pairs and testing for “rate remapping” (Leutgeb et al. 2005). Rate remapping was computed for each cell as the unsigned difference in rates divided by their sum; a paired *t*-test comparison of these values for the same-context and different-context trials was conducted for both peak rates and mean rates. The peak firing rate change was 0.15 Hz for the same-context comparison and 0.16 Hz for the different-context comparisons, and did not differ (*t*_(97)_ = 0.67, NS). The mean firing rate change was 0.16 and 0.14 Hz for the same-context comparison and different-context comparisons, respectively, which likewise did not differ (*t*_(97)_ = −1.20, NS).

**Figure 3.**
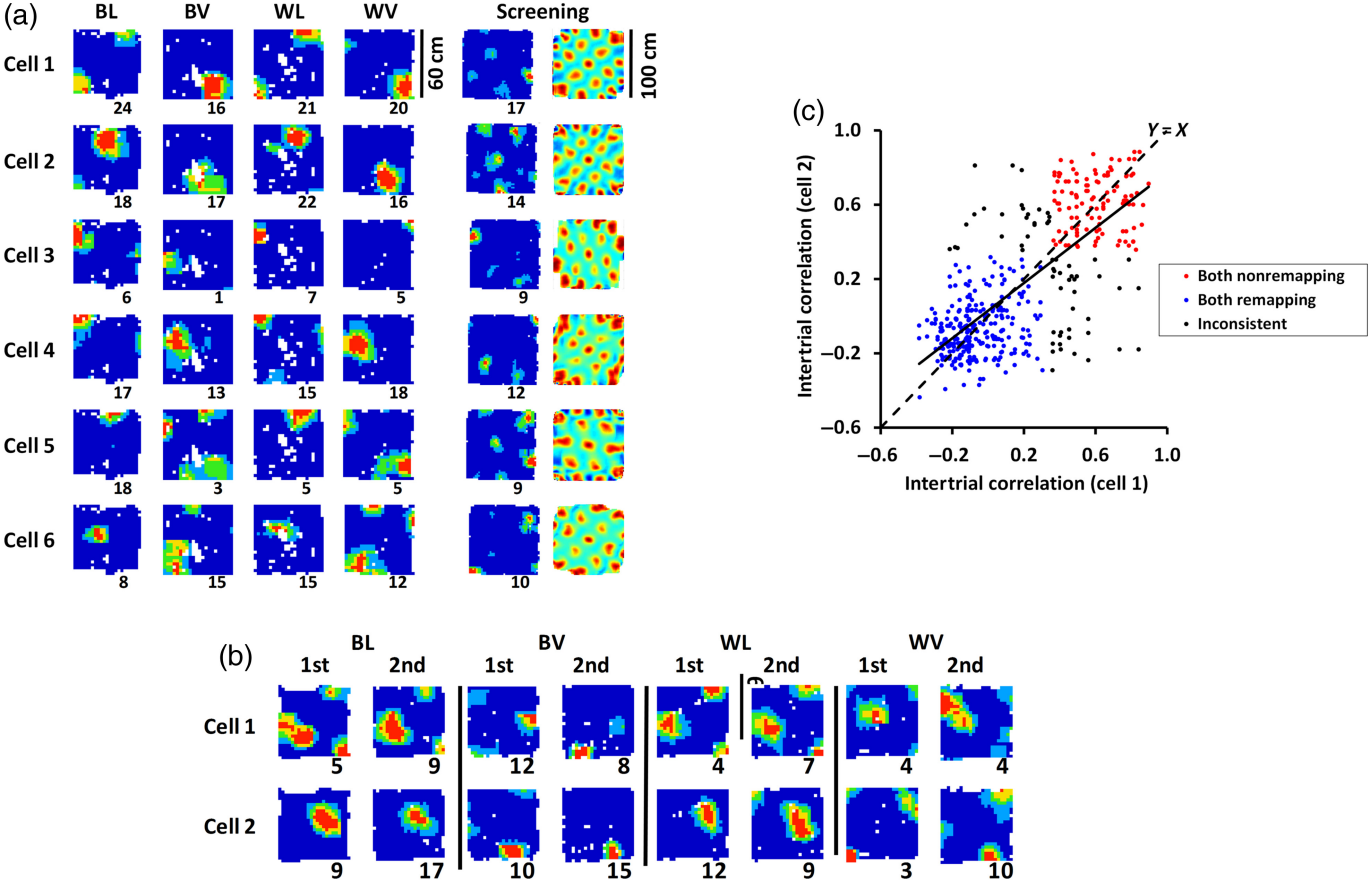
Homogeneity of grid cell responding (*a*) and (*b*) show ratemaps, depicted as in Figure 2. (*a*) Simultaneously recorded grid cells (confirmed by the screening trial ratemaps and autocorrelograms) responding coherently to odor change. (*b*) Two grid cells showing conditional responding (responding to color change only in vanilla context). (*c*) Within-ensemble grid cell pairs responded similarly between context pairs.

In contrast, grid cells showed a distinct change in the position (phase) of their grids in response to context change (Fig. 2*b*). This occurred in response to both color and odor, although it was more pronounced in response to odor (see below). The phase shift was confirmed by correlation of the firing rate maps, which showed high correlation for the within-trial comparison between first and second trial-halves (a check for stability) and also between same-context trials, but showed decreased correlation—realignment—after context change (Fig. 2*c*,*d*). A one-way ANOVA found a main effect of comparison type (i.e., within-trial, same-context, color-change, or odor-change; *F*_3,97_ = 60.00, *P* < 0.0001), with correlations being significantly different between same-context and color-change conditions (same-context correlations = 0.50 ± 0.2, color-change correlations = 0.37 ± 0.2; *t*_(97)_ = 4.42, *P* < 0.0001, and same-context and odor-change conditions (odor-change correlations = 0.15 ± 0.2; *t*_(97)_ = 9.31, *P* < 0.0001). Propensity to realign was greater following odor change than color change (*t*_(97)_ = 6.25, *P* < 0.0001). This was confirmed when realignment was assessed categorically (correlation threshold of 0.35; see Fig. 2*c*); by this criterion, of the 98 cells, 77 responded to odor and 45 to color, which was significantly fewer (*χ*^2^(1, *N* = 98) = 22.23, *P* < 0.0001). Thus, grid cells responded to context changes and were more sensitive to odor change than to color change; indeed, only 8 of these cells responded to color changes alone, while 40 responded to odor changes alone (37 responded to both). Thirty-nine (40%) of the grid cells responded “conditionally” (i.e., response to color change depended on which odor was present, and vice versa; e.g., Fig. 3*b*), indicating convergence of these modalities outside of the cells themselves (Jeffery et al. 2004).

**Figure 4.**
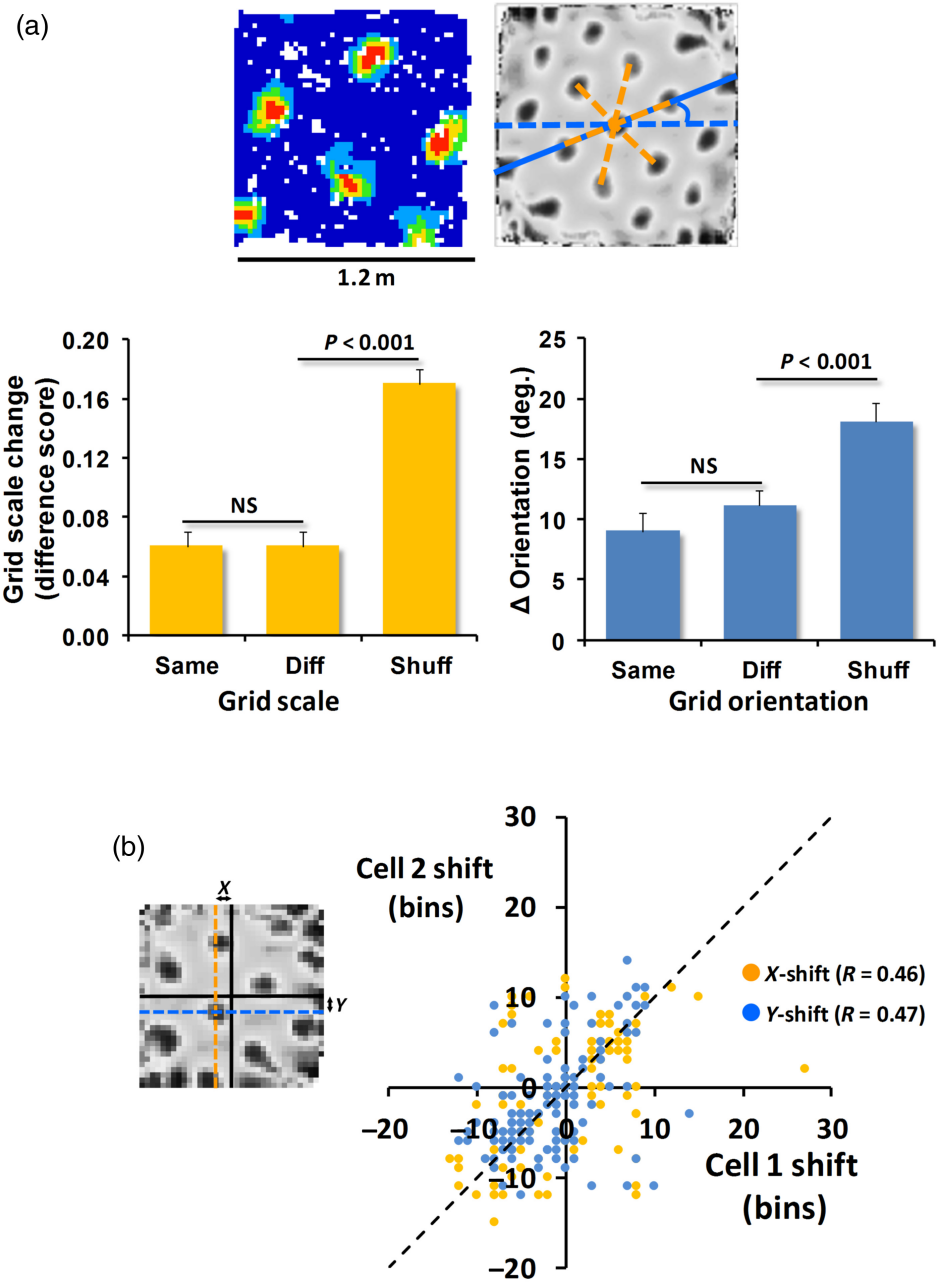
Metric properties of grids (*a*) Top: scale and orientation were measured from the autocorrelograms derived from the ratemaps (scale = orange lines; orientation = blue lines). Bottom: neither grid scale nor orientation differed for same- or different-context comparisons, whereas both differed from random comparisons with shuffled data. (*b*) Left: grid shift was derived from the shift in the central peak of the cross-correlogram in *X* (orange dotted line) and *Y* (blue solid line) from the center (black cross) between context pairs. Right: within-ensemble grid pairs shifted by similar amounts in the *X* and *Y* directions.

A subset of grid cells (*n* = 33) had been recorded in the larger boxes, enabling analysis of metric grid properties of grid scale, orientation, and phase shift from those trials (see Supplementary Methods). The main contextual changes were due to phase shift. We did not find significant changes in grid scale or orientation; grid scale comparison (Fig. 4*a*) between same-cell context pairs and different-cell context pairs (one-way ANOVA on the difference scores) found a significant effect of comparison type (same-cell versus different-cell; *F*_2,32_ = 42.6, *P* < 0.0001) with same-context and different-context pairs not different (*t*_(32)_ = 0.36, NS), but both differing from the shuffled set (same-context versus shuffled; *t*_(32)_ = 6.43, *P* < 0.0001; different-context versus shuffled; *t*_(32)_ = 8.32, *P* < 0.0001). Orientation also did not change (Fig. 4*a*); ANOVA showed a main effect of comparison type [*F*_2,32_ = 7.08, *P* < 0.01] with both the same-context and different-context changes in orientation being small (9° and 11°, respectively) and not different (*t*_(32)_ = 0.95, NS), while both differed significantly from the shuffled set (18°; same-context *t*_(32)_ = 3.16, *P* < 0.01; different-context *t*_(32)_ = 2.85, *P* < 0.01).

Ensemble behavior was examined to see if pairs of simultaneously recorded cells showed similar changes when compared across pairs of contexts, consistent with their presumed attractor dynamics (McNaughton et al. 2006). Twenty-seven ensembles [sizes were 2 (*n* = 17), 3 (*n* = 5), 4 (*n* = 2), 5 (*n* = 3)] yielded a total of 444 cell-pair × trial-pair comparisons, for which ratemap correlations were high within ensembles (*R* = 0.73, *P* < 0.0001). The amount of grid phase shift, obtained from the large-box dataset, was also similar for simultaneously recorded cells (*R* = 0.46 and 0.47 for *x* and *y*, respectively; *P* < 0.0001; Fig. 4*b*).

## Discussion

This study aimed to determine whether grid cells, an important part of the brain network that computes spatial location, respond only to metric spatial information or could also respond to purely nonmetric “contextual” cues manipulated via changes in the color and/or odor of the environment. The same-context paradigm has previously been reported to induce hippocampal place cell remapping (Anderson and Jeffery 2003), and a possible route of this remapping information is via the grid cell network. In the present study, we found a substantial influence of context (especially olfactory context) on the location of cell firing, resulting in grid cells translating their firing fields. It thus appears that grid cells receive nonmetric as well as spatial information, and this information arrives via a different route from the information concerning direction. These findings add to the growing evidence that metric and nonmetric signals remain separated in the spatial navigation system until a late stage in the processing pathway, and suggest that the interaction occurs in the entorhinal–hippocampal network. Interestingly, grid field orientation remained unaffected, and so the manipulation only affected those factors that control grid lateral positioning, and did not affect those that orient it.

As well as examining grid cells in isolation, we examined the ensemble behavior of the cells in order to see whether co-recorded cells showed similar changes, consistent with grid cells being part of an attractor network (McNaughton et al. 2006). Overall grid cells showed that they responded to context in a homogeneous manner (Fig. 3), which differs from the heterogeneous response of simultaneously recorded hippocampal place cells (Anderson and Jeffery 2003). Since we only recorded a few co-localized cells at a time, we cannot rule out that anatomically separated cell assemblies in the entorhinal cortex (so-called modules, which have strong intermodule coupling but weak intramodule coupling; Stensola et al. 2012) respond independently to context change. Thus, the observation of homogeneous realignment in grid cells is preliminary at present. However, it is worth noting that a recent extensive, detailed analysis of grid cell realignment under environmental manipulation found no evidence of inhomogeneous responding (Yoon et al. 2013).

The motivation for the present study was to try to understand better the way in which nonmetric information reaches the place cell system—is it transmitted via the grid cells, or does it arrive independently? The underlying hypotheses are laid out in Figure 1. A previous study by Fyhn et al. (2007) supported the hypothesis in Figure 1*a*—it found that metric environmental change (induced by changing the room or altering the environment from a square to a circle) resulted in entorhinal grid translation and rotation together with complete hippocampal place cell remapping, whereas nonmetric changes (induced by altering the color of environmental walls) caused rate remapping in place cells and no translation/rotation of the grid fields. This finding is consistent with the hypothesis that grid cells transmit context information to place cells via responding (Fig. 1*a*), but did not rule out the possibility that the apparent context sensitivity of grid cells might have been due to their detection of “metric” changes to the environment, leaving open the hypothesis in Figure 1*b* that grids cells are insensitive to purely nonmetric context, and that such signals impinge directly on place cells. Our study shows that grids indeed shift (although they do not rotate) in response to purely nonmetric change and rules out that hypothesis.

Because place cells typically respond heterogeneously to our context paradigm (Anderson and Jeffery 2003), whereas grid cells responded coherently, grid cells look as though they may represent a final decision about context, perhaps received from the place cells, rather than being an intermediate processing stage. However, given the possibility discussed above that different grid cell modules might realign independently, it is possible that anatomically neighboring place cells receive inputs from different grid modules and can thus respond heterogeneously to changes in context. Our findings are thus not inconsistent with the notion that the context signal to place cells is routed through the grid cell network. The reverse is also a possibility however: that is, that context modulates place cells directly and the place cells in turn anchor grid firing fields via back-projections from the entorhinal cortex to the hippocampus (O'Keefe and Burgess 2005; Bonnevie et al. 2013) (Fig. 1*c*). This would be consistent with recent findings that inactivating the hippocampus disrupts grid cell firing (Bonnevie et al. 2013), and that manipulations that heavily attenuate theta band oscillations in the MEC and simultaneously abolish grid field firing do not affect the ability of the hippocampal place cell system to establish and maintain representations in novel environments (Brandon et al. 2014). Further work is needed to establish the direction of contextual information flow.

Stepping back from the grid and place cells, the question arises as to how context information reaches this network from outside. Several investigators have suggested that nonspatial information might be routed via the lateral entorhinal cortex (LEC): for example, Hargreaves et al. (2005) showed that the LEC transmits far less spatial information than does the MEC, suggesting that the LEC might be the pathway for incoming nonspatial information, with convergence occurring in the entorhinal cortex. More recently, Lu et al. (2013) found that responsiveness of CA3 neurons in the hippocampus to color changes in the environment was reduced by LEC lesions. Areas of neocortex surrounding the EC also transmit information about objects, particularly the perirhinal cortex (Deshmukh et al. 2012) and the postrhinal cortex (Furtak et al. 2012), and may be part of a general system whose role is to assemble incoming place-related information to be combined with metric information about distance/direction from borders.

How does the context signal interact with the spatial one to position grids? Some investigators have suggested that the relevant spatial cues are the local boundaries of the environment which could play a role in anchoring the grid pattern (O'Keefe and Burgess 2005; McNaughton et al. 2006; Barry et al. 2007), perhaps acting via border cells or boundary vector cells (Savelli et al. 2008; Solstad et al. 2008). Two recent studies have highlighted the ongoing role of boundaries in constraining grid positioning (Krupic et al. 2015; Stensola et al. 2015). However, border cells are context-insensitive (Solstad et al. 2008; Lever et al. 2009); so why do grid cells shift their grids when the context changes? Our observation suggests that either border cells are not the signal, or else they are not the *only* signal that anchors grid cells to the environment. It may be that context functions to gate the interaction between border cells and grid cells, in a manner akin to the one we have previously proposed for the interaction of contextual and spatial inputs to place cells (Hayman and Jeffery 2008): that is, the context inputs select which of the spatial inputs (mediated via, for example, border cells) gain control of the grid and place cells. Alternatively, perhaps border cells drive place cells directly, and the sensitivity of grid cells to particular border/context combinations derives from the feedback projection from place cells. Untangling the complex causal relationships in this network will ultimately require the application of interventional methods such as optogenetics.

**Figure 5.**
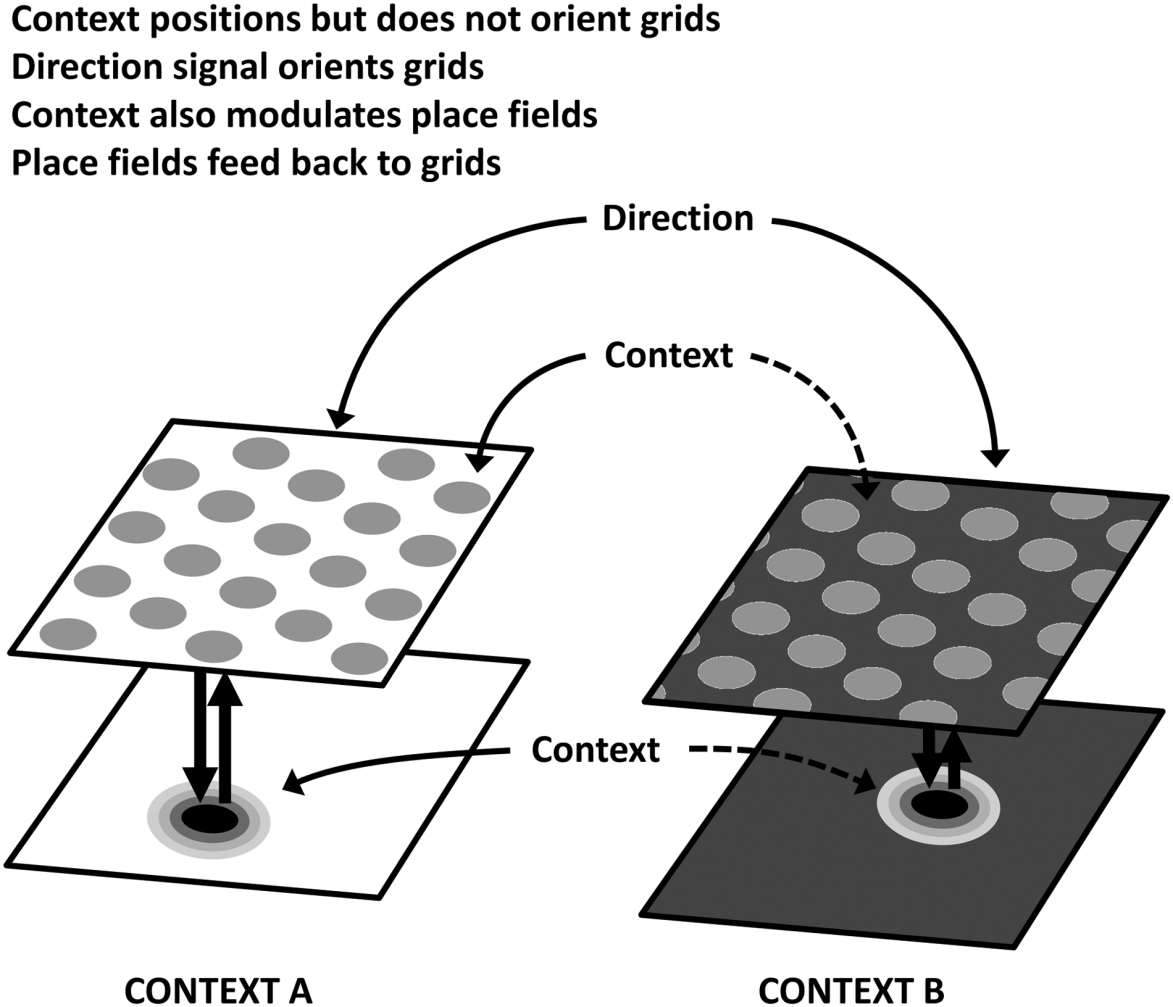
Proposed interaction between contextual and spatial signals. In our proposal, entorhinal grid cells position their grids using combined contextual and spatial inputs and orient the grids using directional information. Contextual inputs also project directly into place cells, which compute a final decision about context which they feed back onto the grid cells.

Putting all this together, our emerging hypothesis is the one illustrated in Figure 5: that nonmetric context information impinges directly onto both grid cells and place cells, but that the grid cell and place cell networks also interact. The final outcome is one in which a complex population code for spatial context is sent out from the place cells, while possibly (with the caveat about modules discussed above) a simpler, homogeneous code is used by the grid cells to position their grids. Whether this positioning has functional relevance for the computing of spatial context, or whether it is an uninformative side effect of contextual remapping in place cells, remains to be determined, and indeed will depend on what function the grid cells turn out to actually have. One possibility is that their function is to support place cells in the temporary absence of external sensory information (e.g., in the dark, or perhaps even simply when attention is diverted), and that under such conditions, the context modulation of grid cells helps them appropriately drive the place cells.

## Supplementary Material

Supplementary material can be found at: http://www.cercor.oxfordjournals.org.

## Funding

The work was supported by studentships from the Biotechnology and Biological Sciences Research Council (BBSRC) to L.L.G. and E.M., and grants to K.J. from the European Commission Framework 7 (Spacebrain), the BBSRC (BB/J009792/1), and the Medical Research Council (G1100669). Funding to pay the Open Access publication charges for this article was provided by the Wellcome Trust.

## Supplementary Material

Supplementary Data
