# Supplementary material for: Purely Translational Realignment in Grid Cell Firing Patterns Following Nonmetric Context Change
Source: Cereb Cortex. 2015 Jun 5;25(11):4619–27. doi: 10.1093/cercor/bhv120 (PMC4816804; doi:10.1093/cercor/bhv120)
Supplement: Supplementary Data [file supp_25_11_4619__index.html]

Purely Translational Realignment in Grid Cell Firing Patterns Following Nonmetric Context Change — Purely Translational Realignment in Grid Cell Firing Patterns Following Nonmetric Context Change — Supplementary Data 

# Purely Translational Realignment in Grid Cell Firing Patterns Following Nonmetric Context Change

## Supplementary Data

Supplementary Data

- Supplementary Data - Doc file
