# Supplementary material for: Purely Translational Realignment in Grid Cell Firing Patterns Following Nonmetric Context Change
Source: Cereb Cortex. 2015 Jun 5;25(11):4619–27. doi: 10.1093/cercor/bhv120 (PMC4816804; doi:10.1093/cercor/bhv120)

**Supplementary figures 1-4** Histological sections at low power (1-2x) and higher power (4.5x) from 16 of the 18 rats (2 brains could not be processed) showing the estimated end-point of the electrode tracks (arrowheads).


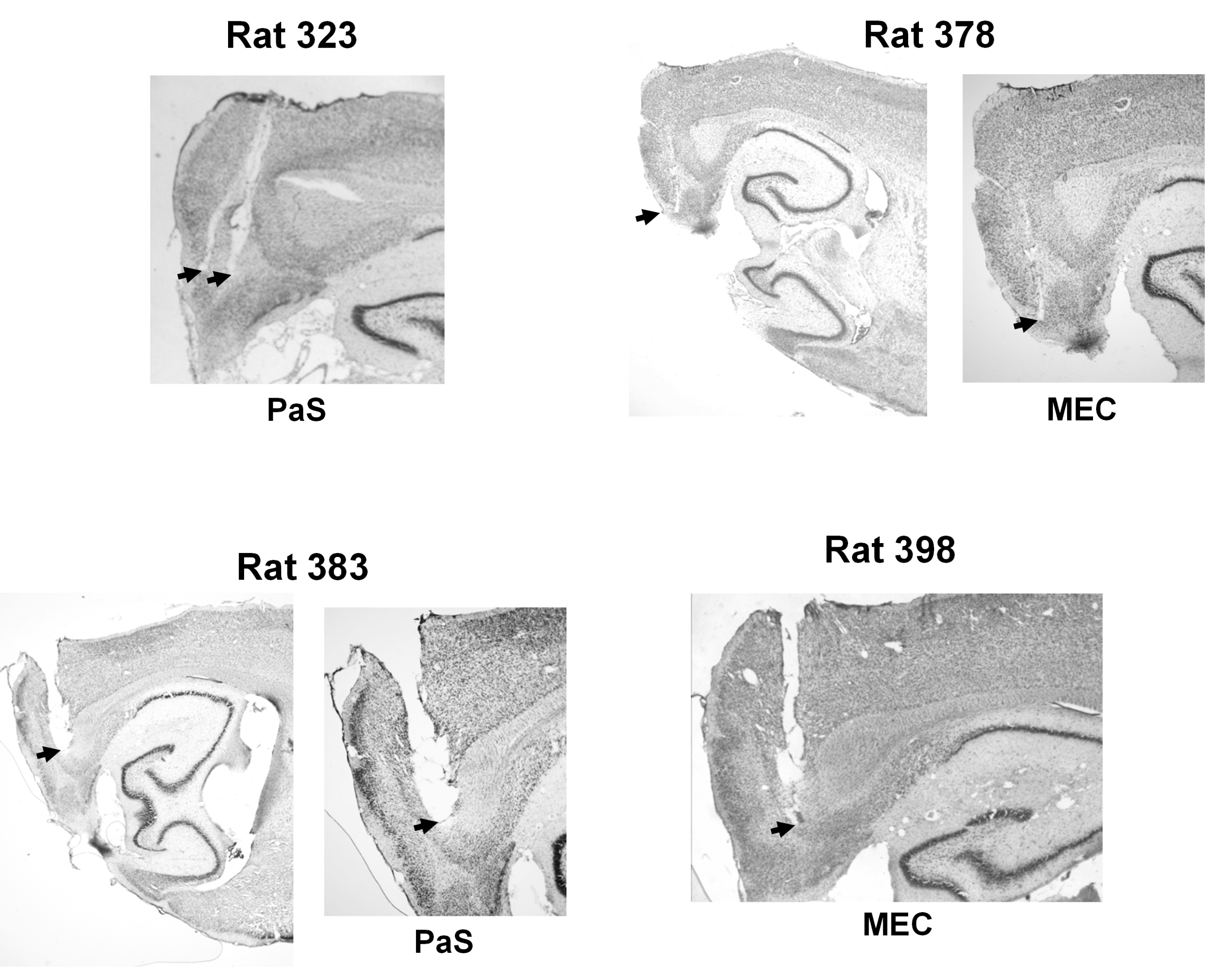


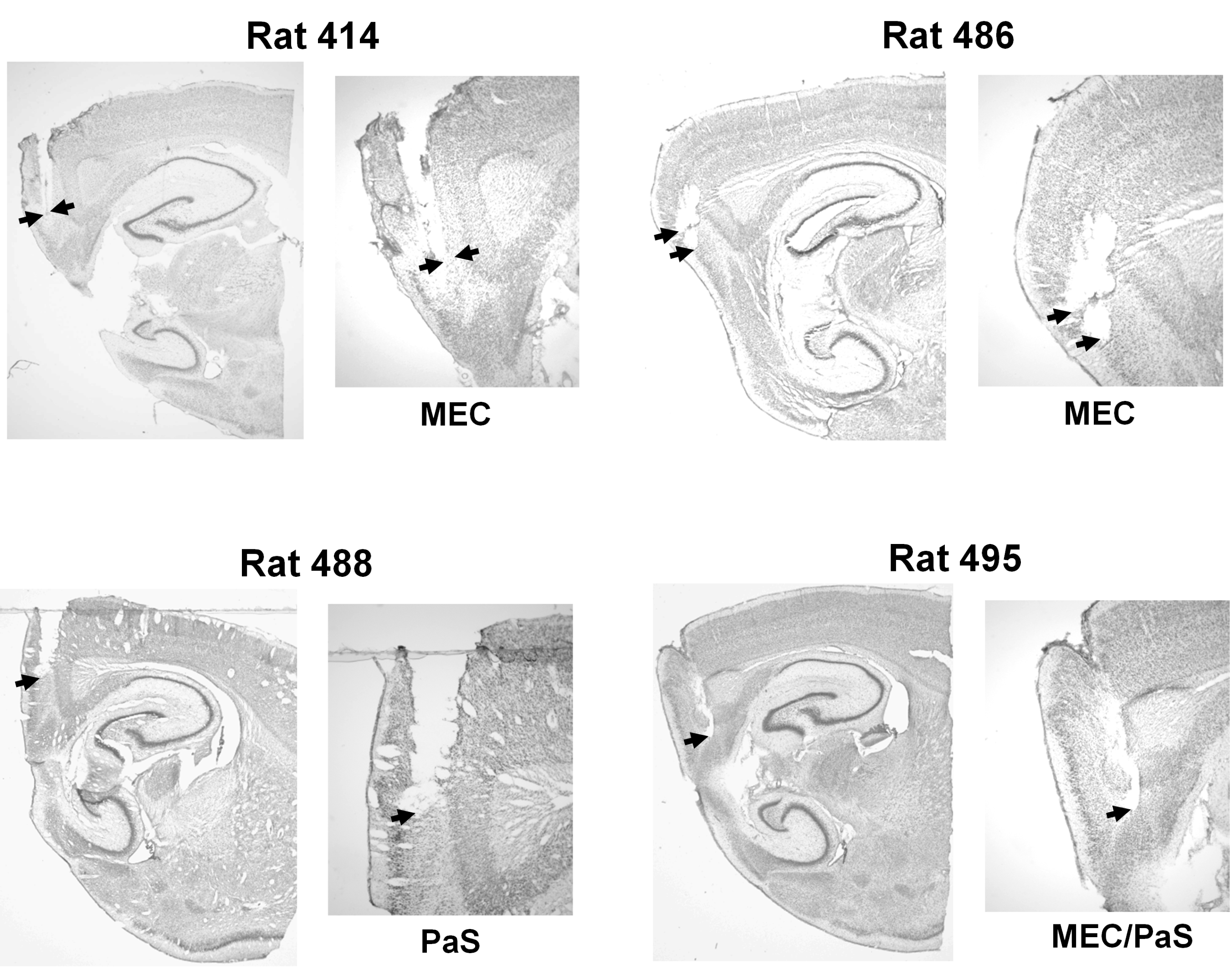


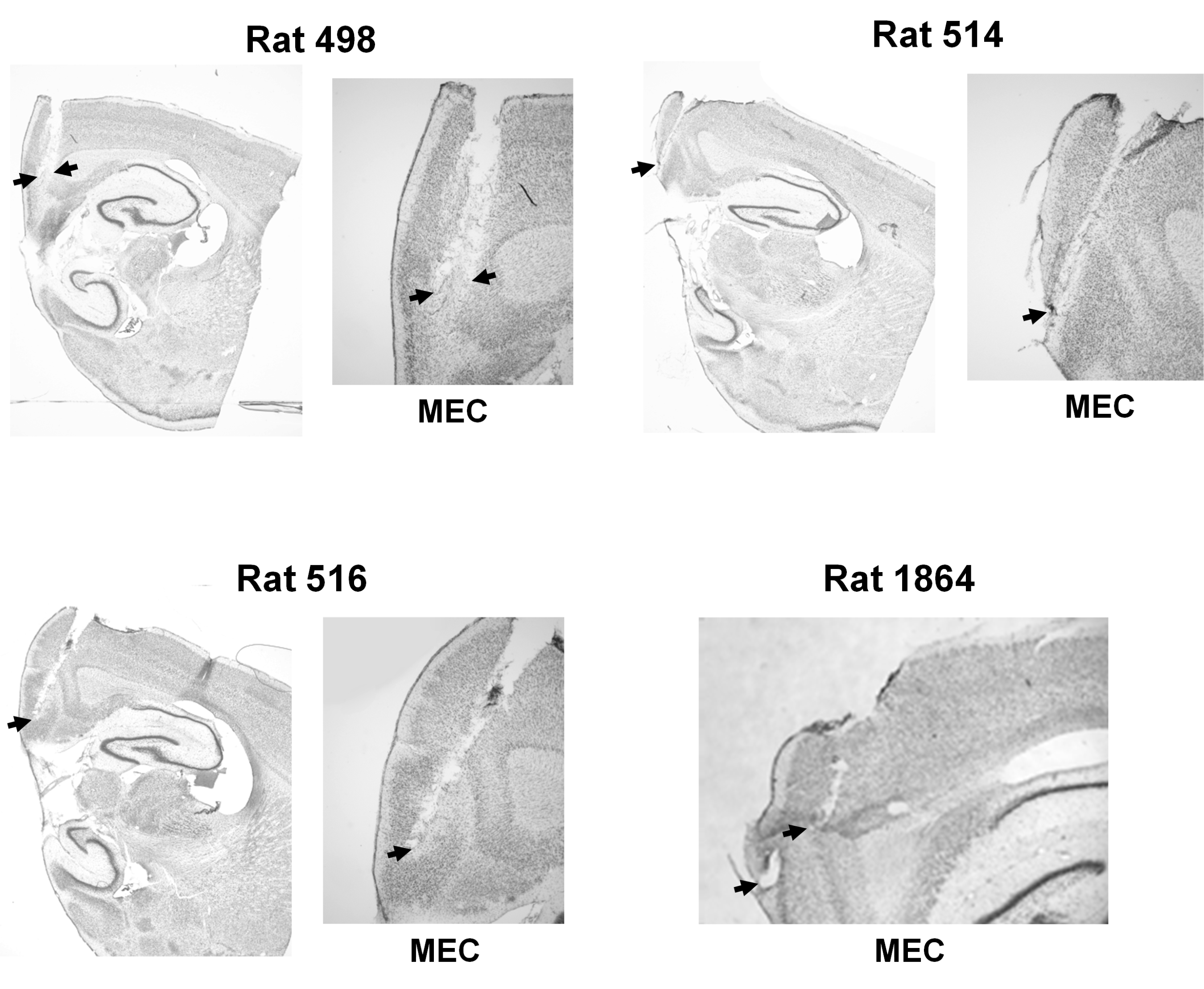


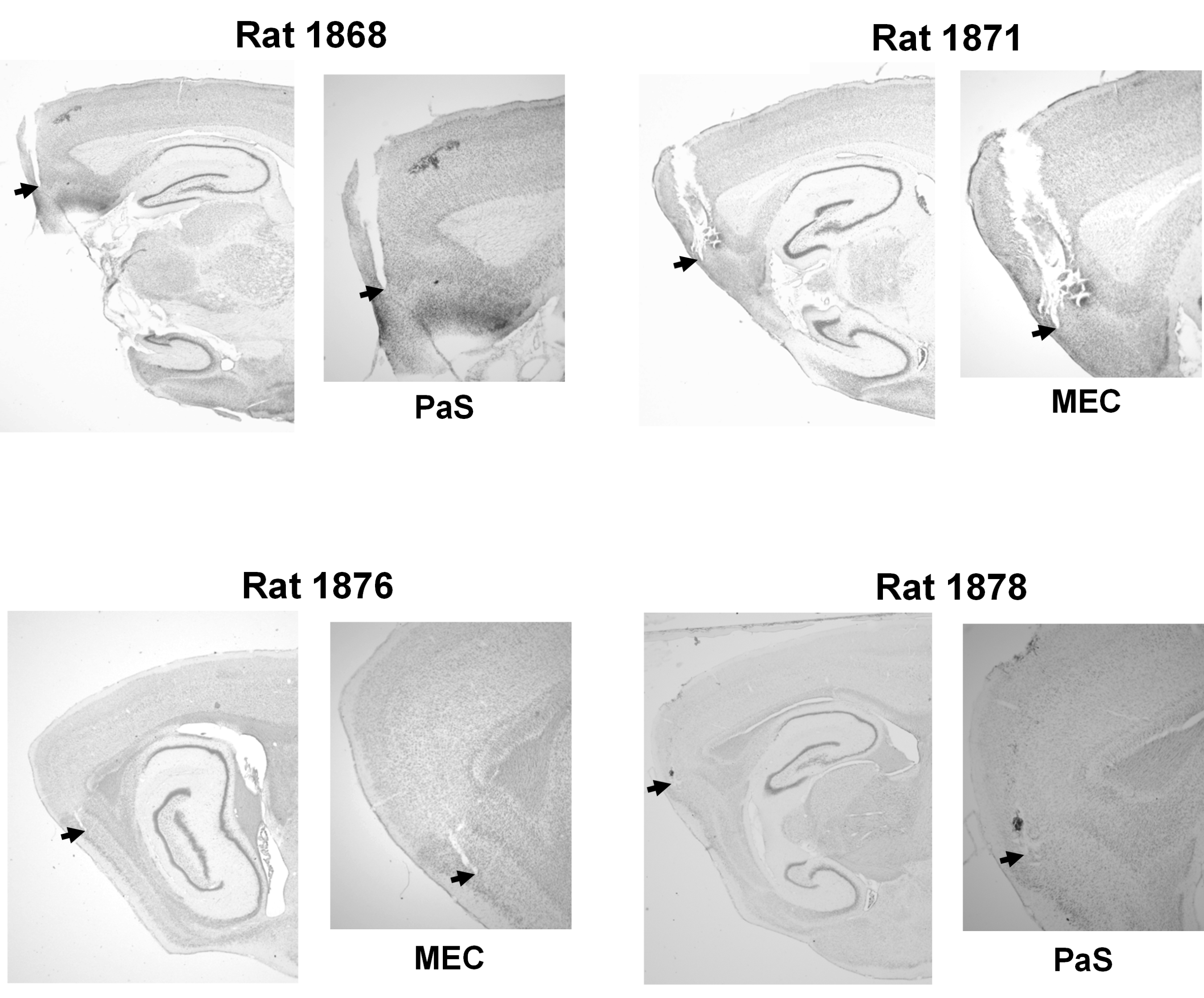


**Supplementary figure 5** Screening trials from each of the rats, illustrating that the electrodes penetrated grid-cell-containing regions


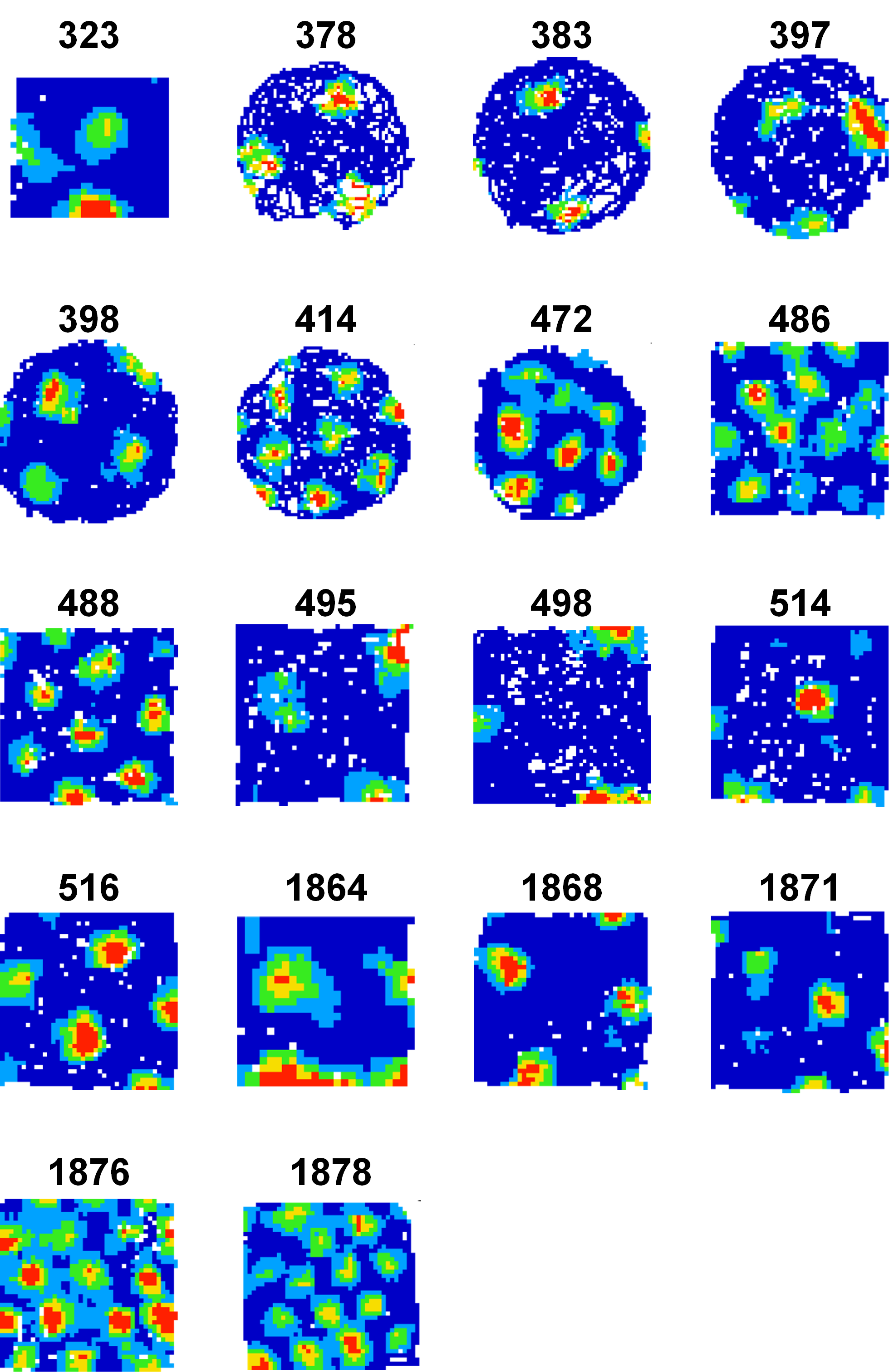

Supplement: Supplementary Data [file supp_bhv120_bhv120supp.doc]
